# Planning implementation and scale-up of physical activity interventions for people with walking difficulties: study protocol for the process evaluation of the ComeBACK trial

**DOI:** 10.1186/s13063-021-05990-3

**Published:** 2022-01-15

**Authors:** Siobhan Wong, Leanne Hassett, Harriet Koorts, Anne Grunseit, Allison Tong, Anne Tiedemann, Colin J. Greaves, Abby Haynes, Andrew Milat, Lisa A. Harvey, Nicholas F. Taylor, Rana S. Hinman, Marina De Barros Pinherio, Matthew Jennings, Daniel Treacy, Sandra O’Rourke, Courtney West, Elizabeth Ramsay, Catherine Kirkham, Claire Morris, Catherine Sherrington

**Affiliations:** 1 Institute for Musculoskeletal Health, The University of Sydney and Sydney Local Health District, Sydney, Australia; 2 Sydney School of Health Sciences, Faculty of Medicine & Health, University of Sydney, Sydney, Australia; 3 School of Exercise and Nutrition Sciences, Deakin University, Burwood, Victoria Australia; 4 Prevention Research Collaboration, Sydney Medical School, Sydney School of Public Health, The University of Sydney, Sydney, Australia; 5 Sydney School of Public Health, The University of Sydney, Sydney, Australia; 6 Centre for Kidney Research, The Children’s Hospital, Westmead, Sydney, Australia; 7 Psychology Applied to Health, School of Sport, Exercise & Rehabilitation Sciences, University of Birmingham, Birmingham, UK; 8 Sydney Medical School, School of Public Health, The University of Sydney, Sydney, Australia; 9 John Walsh Centre for Rehabilitation Research, Northern Clinical School, The University of Sydney, Sydney, Australia; 10 Centre for Sport and Exercise Medicine Research, La Trobe University, Melbourne, Australia; 11 Eastern Health, Alfred Health, Box Hill, Victoria Australia; 12 Centre for Health, Exercise and Sports Medicine, Department of Physiotherapy, The University of Melbourne, Melbourne, Australia; 13 South Western Sydney Local Health District, Sydney, Australia; 14 South Eastern Sydney Local Health District, Sydney, Australia; 15 Flinders University, Adelaide, Australia

**Keywords:** Process evaluation, Implementation, Scale-up, Physical activity

## Abstract

**Background:**

There is currently little evidence of planning for real-world implementation of physical activity interventions. We are undertaking the ComeBACK (Coaching and Exercise for Better Walking) study, a 3-arm hybrid Type 1 randomised controlled trial evaluating a health coaching intervention and a text messaging intervention. We used an implementation planning framework, the PRACTical planning for Implementation and Scale-up (PRACTIS), to guide the process evaluation for the trial. The aim of this paper is to describe the protocol for the process evaluation of the ComeBACK trial using the framework of the PRACTIS guide.

**Methods:**

A mixed methods process evaluation protocol was developed informed by the Medical Research Council (MRC) guidance on process evaluations for complex interventions and the PRACTIS guide. Quantitative data, including participant questionnaires, health coach and administrative logbooks, and website and text message usage data, is being collected over the trial period. Semi-structured interviews and focus groups with trial participants, health coaches and health service stakeholders will explore expectations, factors influencing the delivery of the ComeBACK interventions and potential scalability within existing health services. These data will be mapped against the steps of the PRACTIS guide, with reporting at the level of the individual, provider, organisational and community/systems. Quantitative and qualitative data will elicit potential contextual barriers and facilitators to implementation and scale-up. Quantitative data will be reported descriptively, and qualitative data analysed thematically.

**Discussion:**

This process evaluation integrates an evaluation of prospective implementation and scale-up. It is envisaged this will inform barriers and enablers to future delivery, implementation and scale-up of physical activity interventions. To our knowledge, this is the first paper to describe the application of PRACTIS to guide the process evaluation of physical activity interventions.

**Trial registration:**

Australian and New Zealand Clinical Trials Registry (ANZCTR) Registration date: 10/12/2018.

**Supplementary Information:**

The online version contains supplementary material available at 10.1186/s13063-021-05990-3.

Contributions to the literature

- Despite evidence of the benefits of physical activity, participation rates remain low, particularly for people with a physical disability.
- There is little evidence of implemented and scaled-up physical activity interventions in real-world settings.
- This study will provide insight into potential barriers and facilitators to intervention delivery, implementation and scale-up of the ComeBACK interventions and other similar interventions aimed at increasing physical activity.
- This protocol provides a worked example of how to plan and conduct a process evaluation with a focus on implementation and scale-up.

## Background

The benefits of physical activity participation have been extensively reported, with a comprehensive evidence synthesis supporting the newly updated World Health Organization physical activity guidelines [1]. Despite compelling evidence of the benefits of physical activity and its role in building ‘reserve capacity’ to prevent the decline in physical functioning and independence, participation rates remain low in the general population [2] and are even lower for people with a physical disability [3, 4]. Access to physical activity opportunities can be challenging for people with a disability and is influenced by multiple factors across domains including intrapersonal (e.g. attitudes, beliefs, body functions and structure), interpersonal (e.g. social supports, social processes), institutional (e.g. disability-specific knowledge and processes), community (e.g. education, equipment availability) and policy (e.g. funding, transportation systems) [5]. As such, interventions targeting improvements in physical activity need to be multifaceted, delivered by experienced health professionals, such as physiotherapists [6, 7], and enable tailoring to address and respond to dynamic factors. This can present challenges for evaluation, as traditional methods are more suited to simpler, static, individual-level study designs [8].

When designing and testing interventions, there is an increasing awareness of the need to consider influences on and strategies for translating, disseminating, implementing and scaling up interventions from the beginning [9]. However, there is little evidence of this in most published physical activity research [10, 11]. A recent updated bibliometric review of physical activity research confirms that descriptive physical activity studies continue to dominate the literature, with research relevant for scale-up declining from 28% in 2008/2009 to 17% in 2017/2018 [10]. This supports a previously published literature review where only 3% of papers reviewed reported on outcomes of scaled-up physical activity interventions [9]. Trials of effectiveness are not necessarily designed to capture data on implementation or produce outcomes that are readily transferable to practice. Newer trial designs, such as hybrid designs that consider effectiveness and implementation simultaneously, do so in order to expedite the translation of evidence into practice and policy [12], as well as mediate some of the barriers to translating, disseminating, implementing and scaling up interventions.

Hybrid type 1 studies have a primary focus on effectiveness outcomes whilst using a process evaluation to collect information regarding the implementation experience, adaptations and ongoing supports required [13]. The UK Medical Research Council (MRC) guidance for process evaluations of complex interventions [14] recommends examining implementation (dose, fidelity, adaptions), mechanisms of impact (e.g. participant and stakeholder responses, mediators) and contextual factors to understand the processes through which the intervention affects outcomes. Although this approach will help to inform implementation, it does not necessarily prospectively and systematically consider wider influences on implementation and scalability.

Consequently, we have used the PRACTical planning for Implementation and Scale-up (PRACTIS) guide [15], which provides step-by-step guidance to prospectively and systematically consider factors which may influence intervention implementation and scale-up. The PRACTIS guide recommends an iterative four-step process to identify and plan for barriers and facilitators which can impact effective implementation and scale-up of population interventions. The aim of this paper is to describe the protocol for the process evaluation for the Coaching and Exercise for Better Walking (ComeBACK) randomised controlled trial [16] using the framework of the PRACTIS guide. To our knowledge, this is the first paper to describe the application of PRACTIS to guide the process evaluation of physical activity interventions.

### The ComeBACK trial

The ComeBACK trial is an Australian National Health and Medical Research Council-funded three-arm pragmatic hybrid type 1 randomised controlled trial of community-dwelling adults (*n* = 600) with self-reported difficulty walking. A detailed trial protocol has been published [16] and is briefly described below.

#### Participants

ComeBACK participants are community-dwelling adults with a self-reported difficulty or inability to walk 800 m due to any cause, such as arthritis, neurological impairment or deconditioning. Individuals are ineligible to participate if they report any of the following: are wheelchair dependent; have major cognitive impairment, rapidly progressive neurological disease and insufficient hearing and/or English language skills; are living in residential aged care facilities or those currently meeting the Australian physical activity and sedentary behaviour guidelines for adults [17]. Participants must also have access to a mobile phone (to receive text messages) and Internet access (to use the ComeBACK website). Recruitment is underway in four states in Australia with participants randomised to one of three groups: (i) *Coaching to ComeBACK*, (ii) *Texting to ComeBACK* and (iii) *Texting to ComeBACK Later*.

#### Interventions

**Fig. 1:**
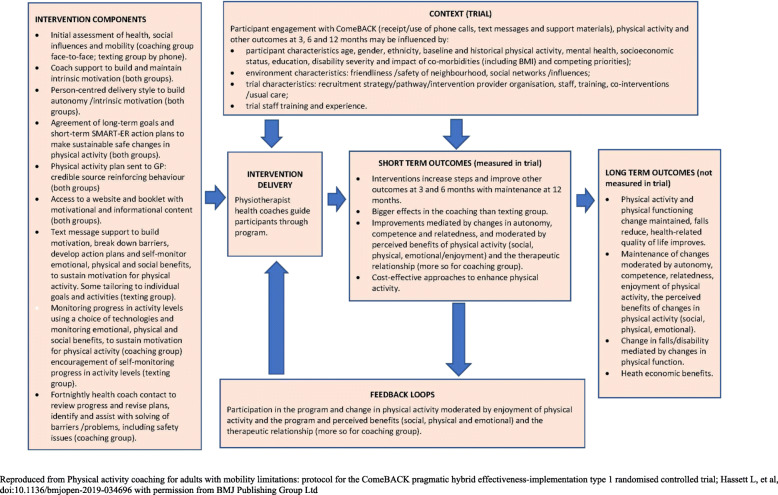
Logic model for the ComeBACK interventions

The ComeBACK interventions vary in intensity and are based on the best current available evidence and theories of behaviour change including the COM-B model [18], Self-Determination Theory [19], Social Cognitive Theory [20] and Self-Regulation Theory [21] as described in the trial logic model (Fig. 1).

The components of each intervention are described in detail in Table 1. Briefly, they consist of the following: (i) *Coaching to ComeBACK*: a physiotherapy assessment of physical capacity; handover between participant, health coach and physiotherapist; and fortnightly tailored telephone health coaching sessions by a physiotherapist. Participants can also access technologies such as pedometers, activity monitors or physical activity smartphone apps if desired; (ii) *Texting to ComeBACK:* a single telephone call by a physiotherapist health coach and text messages with some personalisation and tailoring at a frequency of 5 times per week initially with an option to alter the frequency of text messages after 1 month. The wait list control group, *Texting to ComeBACK Later*, receive the *Texting to ComeBACK* intervention after a 6-month delay.

**Table 1:** Components of the ComeBACK interventions

ComeBACK interventions	
*Coaching to ComeBACK*	Physiotherapy assessment of mobility status, safety issues, medical, social and environmental influences on mobility, delivered face-to-face or via telephone/videoconference.
	Handover phone or video conference between participant, health coach and physiotherapist to understand the participant’s capacity and environment prior to the health coaching intervention.
	Fortnightly tailored telephone health coaching sessions by a physiotherapist with experience in the management of people with walking difficulties, incorporating goal setting, problem-solving, building social support, experiential learning and motivational interviewing.
	Participants also have access to technologies such as pedometers, activity monitors or physical activity smartphone apps if desired.
*Texting to ComeBACK/Texting to ComeBACK Later*	A single telephone call by a physiotherapist health coach with experience in the management of people with walking difficulties. The health coach provides tailored advice based on information from the baseline assessment of capability, identifying appropriate physical activity opportunities and building motivation.
	Participants then receive text messages with some personalisation and tailoring at a frequency of 5 times per week over the first month. They can then elect to increase (daily messages) or decrease (3 messages/week) the frequency of text messages for 3 months before there is a reduction in messages (1–4 messages/week) for the remaining 2 months of the intervention period. There is an ‘opt out’ feature available to participants at all times.
*All groups*	Paper and Web-based educational information regarding the benefits of physical activity, strategies on how to overcome barriers to increasing physical activity and video case studies to model how others have achieved this.
	Physical activity plan that is developed in conjunction with the health coach on their initial telephone call. This is also shared with the participants’ general practitioner to increase awareness of the intervention and enable discussion and reinforcement of the benefits of physical activity.

Reproduced from Physical activity coaching for adults with mobility limitations: protocol for the ComeBACK pragmatic hybrid effectiveness-implementation type 1 randomised controlled trial; Hassett L, et al, doi:10.1136/bmjopen-2019-034696 with permission from BMJ Publishing Group Ltd

All participants are provided with paper and Web-based educational information and case studies (after 6 months for the *Texting to ComeBACK Later group*) and a personalised physical activity plan developed with the health coaches and shared with the participants’ general practitioner (GP), i.e. local doctor. The ComeBACK interventions are delivered over a 6-month period, with final follow-up at 12 months post-randomisation.

Ethical approval for the ComeBACK trial and process evaluation was granted by the lead Ethics Review Committee (Royal Prince Alfred Hospital Zone) of the Sydney Local Health District (Protocol No. X18-0234) with site-specific ethical approval obtained for all individual recruitment sites. Written informed consent is obtained from participants prior to their involvement in the study. All reported study data will be de-identified.

## Methods

### Design

**Fig. 2:**
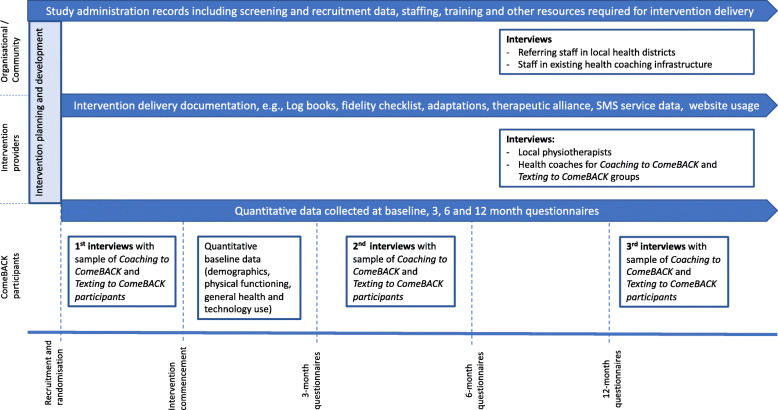
ComeBACK process evaluation, data collection and timeline

A mixed methods process evaluation has been embedded within the ComeBACK trial and is informed by the UK MRC guidance on process evaluation of complex interventions [14] and structured using the PRACTIS guide [15]. Figure 2 provides an overview of the process evaluation. The application of the PRACTIS guide within the ComeBACK trial process evaluation is described in detail below.

#### PRACTIS step 1: Characterise the parameters of the implementation setting

This includes considering the needs of people involved and what resources are necessary to deliver the interventions.

##### Target population

The target population (described previously) is recruited from health services in four states in Australia (via study-supported research assistant, the treating health professional or ComeBACK brochures/posters at recruitment sites) or from the general community across Australia (via advertising in print and digital media including social media). Recruitment is monitored using a logbook to capture costs and insights about methods for engaging this population in the future.

##### Implementation staff

The health coaches delivering ComeBACK interventions are registered physiotherapists with experience in the management of people with walking difficulties and in delivering telephone health coaching in other research trials [22, 23]. They received training in behaviour change techniques (including motivational interviewing) and intervention delivery processes to standardise the intervention delivery. Coaches delivering the intervention are current employees of the institute where the research is being conducted and receive mentoring from study investigators and an external provider with extensive health coaching experience. These details are recorded as administration data in a logbook and will be reported descriptively along with staffing costs to provide details on implementation staff requirements for future implementation.

##### Resources

Most of the ComeBACK interventions can be delivered remotely from a centralised location with resources such as a computer, telephone and Internet access. The face-to-face element of the interventions, i.e. the assessment of physical capacity and mobility within the *Coaching to ComeBACK* group, has been modified to video conferencing/phone calls, where necessary, as a result of restrictions due to the COVID-19 pandemic. The effects of this modification will be discussed with health coaches and participants in semi-structured interviews described below. Where a physiotherapist has conducted a face-to-face assessment, details such as where they were sourced and costs involved are recorded.

Discrete parts of the ComeBACK interventions, such as the Web-based Short Messaging Service (SMS) delivery service and website development, have been contracted to organisations with existing relationships with health services to facilitate future embedding of the interventions into existing infrastructure. Additional resources, such as paper-based educational material and physical activity monitors, are also required for intervention delivery. All resources and services required to set up and deliver the ComeBACK intervention will be logged on an MS Excel spreadsheet to identify and value costs.

#### PRACTIS step 2: Identify and engage key stakeholders across multiple levels within the delivery system

Early engagement of key stakeholders is likely to lead to better partnerships, involvement and long-term sustainability of the programme. Key stakeholders include (i) those that fund or have ownership of the interventions, (ii) those responsible for the dissemination of the interventions to the target setting and population, (iii) those delivering the interventions and (iv) the target population receiving the interventions. For the ComeBACK trial, these stakeholders were engaged during intervention development. Previous qualitative work [23, 24] conducted with end users regarding their experiences of health coaching and technology use in rehabilitation has informed ComeBACK intervention development. Health service managers and government policymakers were engaged, at various stages, in the intervention design and are investigators in the trial.

#### PRACTIS step 3: Barriers and facilitators to implementation and scale-up

Identifying the potential barriers and facilitators to implementation and scale-up at an early stage may enhance the integration of research findings into real-world settings. It can also identify aspects of intervention design and implementation/scale-up planning that may require refinement.

A detailed plan for monitoring and collection of qualitative and quantitative data (Table 2) was developed to capture the potential barriers and facilitators to implementation and scale-up of the ComeBACK interventions across the various levels of the delivery system (i.e. individual, provider, organisational and community/system). This is described in more detail below. All qualitative work will be conducted by a postgraduate researcher (SW) under the guidance of experienced qualitative researchers (AT, AH).

**Table 2:** PRACTIS step 3: data on potential barriers and facilitators to implementation of the ComeBACK interventions collected as part of the ComeBACK process evaluation

Potential barriers and facilitators to implementation of the ComeBACK interventions				
***Individual level***	**Potential barriers and facilitators**	**Data source**	**Collected from**	**Time**
Characteristics of the participants	Age, sex, ethnicity, mental health, socioeconomic status, education	Baseline demographic data and Warwick-Edinburgh Mental Wellbeing Scale	All groups	Baseline
Capability to participate in the ComeBACK interventions	Baseline level of mobility, physical activity and co-morbidities	Lower Limb Extremity Function and Disability questions; Walking capacity and use of aids, Incidental and Planned Exercise Questionnaire (IPEQ), Functional co-morbidity index	All groups	Baseline
	Baseline level of technology use	Technology exposure survey	All groups	Baseline
	Baseline pain in lower limbs	Pain-related questions and score	All groups	Baseline
	Falls history and balance confidence	Falls history and fear of falling	All groups	Baseline
	Self-efficacy to participate in the programme	Impressions of the program questionnaire Q5, 8	All groups	Gp 1 and 2—6mths Gp 3—12mths
	Confidence in ability to be physically active	Semi-structured interviews Coaching: Int1 Q1; Int2 Q6 and 10; Int3 Q5 Texting: Int1 Q1; Int2 Q5 and 8; Int 3 Q5	Sample from Coaching to ComeBACK and Texting to ComeBACK groups	Int1: post-randomisation, prior to commencement of the intervention Int2: 4–6mths post-randomisation
	Problem solving skills (e.g. experiential learning)	Semi-structured interviews with participants Int1 Q5 Int2 Q11 Int3 Q3	Sample from Coaching to ComeBACK and Texting to ComeBACK groups	Int1: prior to commencement of the intervention Int2: 4–6mths post-randomisation Int3: 9–12mths post-randomisation
Opportunity available to participate in the ComeBACK interventions	Suitable and affordable local opportunities to engage in physical activity (e.g. community-based programmes; home exercise programmes; website links sent regarding physical activity opportunities)	Health coach data logs	Coaching to ComeBACK	Continuous
		Impressions of the program questionnaire Q6	All groups	Gp 1 and 2—6mths Gp 3—12mths
		Semi-structured interviews with participants Coaching: Int2 Q7 Texting: Int2 Q6	Sample from Coaching to ComeBACK and Texting to ComeBACK groups	Int2: 4–6mths post-randomisation
	Access to resources, including technology, if desired.	Semi-structured interviews with participants Coaching: Int2 Q9 Texting: Int2 Q7	Coaching to ComeBACK	Int2: 4–6mths post-randomisation
		Impressions of the program questionnaire Q4	All groups	Gp 1 and 2—6mths Gp 3—12mths
	Changes in the environmental context impacting on opportunities to be physically active (e.g. COVID-19; bushfires)	Semi-structured interviews with participants in response Coaching: Int1 Q8; Int2 Q1; Int3 Q1 Texting: Int1 Q9; Int2 Q1; Int3 Q1	Sample from Coaching to ComeBACK and Texting to ComeBACK groups	Int1: post-randomisation, prior to commencement of the intervention Int2: 4–6mths post-randomisation
		Impressions of the program questionnaire Q10	All groups	Gp 1 and 2—6mths Gp 3—12mths
		Informal feedback from participants via text message reply/emails to team/written letters/health coaching logs	All groups	Continuous
Motivators to engage in the ComeBACK interventions	Acceptability of the interventions	Health coach data logs	Coaching to ComeBACK	Continuous
		Uptake and usage of activity monitors as recorded by health coaches in data logs	All groups	Continuous
		Semi-structured interviews Coaching: Int2 Q1, 2, 6, 7, 8, 9; Int3 Q6 Texting: Int2 Q1, 2, 5, 6, 7, 9, 10; Int3 Q6	Sample from Coaching to ComeBACK and Texting to ComeBACK groups	Int2: 4–6mths post-randomisation Int3: 9–12mths post-randomisation
		Rating the components of the intervention via the impressions of the program questionnaire Q1, 2, 3, 4, 7	All groups	Gp 1 and 2—6mths Gp 3—12mths
		ComeBACK website usage data from Google analytics (number of visits; pages visited; time spent on site)	All groups	Continuous
		Text message delivery data from Web-based text messaging service (alterations in frequency of delivery or opt out)	Texting to ComeBACK and Texting to ComeBACK Later	Continuous
		Informal feedback from participants via text msg reply/emails to team/written letters	All groups	Continuous
		Withdrawal reasons reported in REDCap	All groups	Continuous
	Enjoyment of the interventions	Physical Activity Enjoyment Scale (PACES)	All groups	Baseline, 3mth, 6mth and 12mths
		Experiences related to physical activity questionnaire	All groups	3mth, 6mths and 12mths
		Impressions of the program questionnaire Q1, 2, 7, 8, 10	All groups	Gp 1 and 2—6mths Gp 3—12mths
	Therapeutic alliance	Working Alliance Inventory-Short Report (participant)	All groups	Gp 1 and 2—6mths Gp 3—12mths
		Semi-structured interviews with participants Coaching: Int2 Qs 4, 5 Texting: Int2 Qs 4, 5	Sample from Coaching to ComeBACK and Texting to ComeBACK groups	Int2: 4–6mths post-randomisation
	Attitude to physical activity	Attitudes to physical activity questionnaire	All groups	Baseline, 3mths, 6mths and 12mths
***Provider level***	**Potential barriers and facilitators**	**Data source**	**Collected from**	**Time point**
Capacity of providers to deliver the ComeBACK interventions effectively	Provider experience, training and ongoing support/mentorship necessary to deliver interventions to this population safely and effectively.	Interview with health coaches Int Q1	Health coaches	Toward the end of the trial period
		Training log	Training log	Ongoing
		Minutes from meetings with investigators and other health coaches engaged for supervision	Health coaches	Continuous
	Feasibility of a one-off physiotherapy assessment	Semi-structured interviews with local physiotherapists Int Q4, 5, 6, 7, 8, 9 and health coaches Int Q8, 9	Local physiotherapists and health coaches	Toward the end of the trial period
	Feasibility of providing physical activity advice without a physical assessment in this population	Interview with health coaches Int Q9	Health coaches	Toward the end of the trial period
	Therapeutic alliance	Work Alliance Inventory-Short Revised (Therapist)	All groups	Health coaches Gp 1—6mths Gp 2 and 3—after initial telephone call of tailored advice (approx. 2 weeks)
		Interview with health coaches Int Q16	Health coaches	Toward the end of the trial period
	Understanding the providers preconceived biases of the effectiveness of the interventions	Interview with health coaches Int Q4, 5	Health coaches	Toward the end of the trial period
Can the interventions be delivered with fidelity?	One-off physiotherapy assessments are conducted with fidelity	Health coach data logs and Physiotherapy assessment forms	Coaching to ComeBACK	Continuous
	Fortnightly health coaching sessions (frequency, duration, behaviour change content)	Health coaching data logs Checklist sample (10%) of content	Coaching to ComeBACK	Continuous
	One-off phone call of tailored advice (frequency duration and content)	Texting data logs Checklist sample (10%) of content	Texting to ComeBACK Texting to ComeBACK Later	Continuous
	Text messages delivered	Online SMS delivery service log	Texting to ComeBACK Texting to ComeBACK Later	Continuous
	Physical activity plans developed and sent to the general practitioner/local doctor	Review log of physical activity plans sent to general practitioner/local doctor	All groups	Continuous
Provider perceptions on how the interventions can overcome the common barriers to physical activity identified by this population	Are the providers able to work with the commonly reported barriers to physical activity in this population?	Interview with health coaches Int Q 2, 3, 16	Health coaches	Toward the end of the trial period
***Organisational level***	**Potential barriers and facilitators**	**Data source**	**Collected from**	**Time point**
Implementation of the interventions	Ability to engage potential participants in the ComeBACK trial	Screening and recruitment databases	Trial admin	Continuous
		Advertising log	Trial admin	Continuous
		Staffing profile required for recruitment	Trial admin	Continuous
		Qualitative interviews/focus groups with other stakeholders	Staff from the allied health clinicians, health service managers, health promotion units	Throughout trial period
	Integration of the interventions into current healthcare practices?	Impressions of the program questionnaire Q9	All groups	Gp 1 and 2—6mths Gp 3—12mths
Sustainability of the intervention	Costings of setting up and delivering the intervention	Economic analyses of the ComeBACK trial*	All study groups and processes	Throughout trial period
	Adaptations required during intervention delivery (e.g. follow up to text messages; tech support, email to participants)	Health coaching and texting data logs; reply messages received; phone calls received	All groups	Continuous
	Staffing profile and resources necessary to set up and deliver the interventions, and any additional tasks or costs to deliver the intervention	Resources and training log	Trial admin	Continuous
	Accessibility of required resources within existing health infrastructure	Resources utilised that have existing relationships with Health departments, e.g. SMS delivery service	Trial admin	Continuous
		Qualitative interviews/focus groups with stakeholders	Allied health clinicians, health service managers, health promotion units, general practitioner/local doctor	Toward the end of the trial period
***Community/systems level***	**Potential barriers and facilitators**	**Data source**	**Collected from**	**Time point**
Dissemination of the programme	Dissemination of ComeBACK intervention by community and government	Semi-structured interviews or focus groups with staff from existing coaching infrastructure (e.g. *Get Healthy NSW*) and government policymakers re. implementation and scale-up of the ComeBACK interventions	Other stakeholders	Throughout trial period
	Scope of the ComeBACK interventions to fit into existing infrastructure	Comparison between existing infrastructure (e.g. Get Healthy NSW) and requirements of the ComeBACK interventions	Collated information from trial processes and stakeholder interviews	Throughout trial period

*Int* interview, *Gp* group, *PA* physical activity, *mth/s* month/s, *SMS* short messaging service, *Gp 1* Coaching to ComeBACK, *Gp 2* Texting to ComeBACK, *Gp 3* Texting to ComeBACK Later

*Economic evaluation will be conducted independently of this process evaluation

#### Individual level

##### Qualitative data

Semi-structured telephone interviews with 15–20 participants from each of the *Coaching to ComeBACK* and *Texting to ComeBACK* groups will explore participant expectations, motivation, self-efficacy and other barriers and enablers of both physical activity and participation in the programme. Interviews will be conducted at three time points across the course of the trial period: (1) prior to commencing the intervention; (2) 4 to 6 months after commencement of the intervention and (3) after the completion of the intervention at 9 to 12 months. Interviews take between 30 and 40 min. The interview guide is available in Additional file 1.

Participants will be purposively sampled for maximum variation in age, sex, extent of impaired mobility and recruitment source [25]. Data collection and analysis will occur in parallel and continue until thematic data saturation is achieved [26]. That is, no new concepts or themes arise from subsequent interviews and there are data of sufficient quality to inform the research questions.

##### Quantitative data

For trial participants, demographic characteristics (age, sex, ethnicity, socioeconomic status and education) as well as physical functioning (mobility, physical activity and falls history), general health (co-morbidities, mental health and pain) and technology use will be collected via questionnaires prior to randomisation to describe participant characteristics in relation to retention and outcome variables.

Participants’ perceptions of acceptability will be assessed using a study-specific questionnaire (*Impressions of the program*) completed post-intervention (Additional file 2). Participants’ enjoyment of the interventions is captured using the Physical Activity Enjoyment Scale (PACES) [27] and attitudes to and experiences of physical activity collected via a study-specific survey at 3 months, 6 months and 12 months. Therapeutic alliance—the co-operative working relationship between participant and health coach—will be measured using the Working Alliance Inventory—Short Revised (participant version) [28] at the end of the intervention period.

The health coaches providing the ComeBACK interventions maintain logs of their contact with participants including details of the frequency and duration of health coaching calls, community exercise opportunities available and technology used. They also record participants’ usage of activity monitors such as Fitbits to understand the uptake of such devices throughout the trial period. The dose of text messages received and the number of participants who increase, decrease or opt out of the messaging are captured by the Web-based SMS service. Google analytics is used to track the activity on the intervention websites including the number of visits, pages viewed and time spent on the site.

Changes in the environmental context which may impact opportunities available to be physically active (such as the January 2020 bushfires affecting parts of Australia and COVID-19 pandemic) are being recorded by study staff as additional potential influences of engagement in the ComeBACK interventions. All informal feedback, such as contact through emails, letters and text message replies are being collated.

#### Provider level

##### Qualitative data

The two health coaches will be invited to participate in a joint interview to facilitate the exchange and development of ideas. An interview guide has been developed with the research team to explore their expectations of the interventions, thoughts on the mechanisms of impact, barriers and enablers in delivery and potential for implementation and scale-up. This interview will take approximately 60–90 min and will occur toward the end of the intervention delivery period. The interview guide is available in Additional file 1.

Semi-structured 20–30-min telephone interviews will be conducted with local physiotherapists who have completed the one-off assessment with *Coaching to ComeBACK* participants. These interviews will investigate the model’s viability for implementation at scale and identify any barriers or enablers. Physiotherapists (*n*~8–10) will be purposively sampled for maximum variation in geographical location.

##### Quantitative data

Quantitative data on provider-level influences will be collected from numerous sources during the trial. These sources include log workbooks completed by the health coaches and study staff. For example, evidence of training, support and mentorship of the health coaches, and relevant meeting minutes with investigators to discuss behaviour change strategies and brainstorm challenging cases will be documented through a training log.

Multiple methods will be used to assess the fidelity of intervention delivery. The physiotherapy assessment forms in the *Coaching to ComeBACK* group will be reviewed for data consistency, quality of content and completeness including the objective measurement(s) of functional capacity, physical impairments assessed and information on social and environmental status. The health coaching and texting logs will also be reviewed for frequency and duration of sessions. Reports from the Web-based text message service will provide details on the number of messages delivered to each participant. In addition, a random sample of telephone calls to participants in the *Coaching to ComeBACK* and *Texting to ComeBACK* groups will be audited using an intervention delivery fidelity checklist to review the behaviour change techniques employed. This checklist has been developed based upon a revised taxonomy of behaviour change techniques specifically aimed at increasing physical activity and healthy eating [29]. The health coaches and study investigators discussed items on the checklist and agreed on a finalised version reflective of the behaviour change techniques employed during the intervention delivery for this population. The checklist will be completed by SW, who will be present during a random sample (*n* = 20) of health coaching calls to participants in the *Coaching to ComeBACK* and *Texting to ComeBACK* groups. The number of physical activity plans forwarded to local doctors will be monitored to review adherence to the intervention protocol.

#### Organisational level

To further understand the barriers and facilitators for reaching and engaging with the target population if implementation and scale-up were to occur, we will conduct semi-structured interviews and/or focus groups with health professionals working in the health services that recruit for the study. This includes professionals with direct contact with potential participants, such as physiotherapists, or those who may be involved in future recruitment (such as health promotion staff) or responsible for decisions about implementation and scaling these types of interventions (health service managers and other decision-makers within the healthcare system). Their experiences, thoughts and attitudes towards implementing interventions like ComeBACK in the Australian healthcare context will be sought. It is envisaged that the focus groups and/or semi-structured interviews may be face-to-face or via video conference/telephone, depending on the availability and preference of the interviewee, and take between 30 and 60 min depending on the format required. The interview guide will be available in Additional file 1.

#### Community/systems level

In terms of the barriers and facilitators to implementation and scale-up of these interventions in the context of the community and wider systems, we will explore how the ComeBACK interventions can be integrated and work across existing health systems and the community in different states. We will have gathered some of this information in aforementioned focus groups and/or semi-structured interviews with stakeholders including participants, healthcare clinicians and managers, as well as health promotion staff within health services. We also plan to invite staff working at existing telephone health coaching services (e.g. *Get Healthy NSW*) to explore the model of service delivery and how the ComeBACK interventions may integrate with them. The semi-structured interviews may be face-to-face or via video conference/telephone, depending on the availability and preference of the interviewee, and would likely take between 30 and 40 min. The interview guide will be available in Additional file 1.

It is recognised that adaptation, a process of deliberate alteration to the design or delivery of an intervention, is a key concept in implementation [30]. Adaptations may be proactive or reactive, considering the intent or goal of the modification, as well as contextual factors which may influence the decision. As such, all adaptations and modifications to the ComeBACK interventions and intended delivery will be recorded by study staff using the Framework for Modification and Adaptations—Expanded (FRAME) [31].

### Data analysis

Data, such as Working Alliance Inventory—Short Revised [28], PACES [27] and the attitudes to and experiences of physical activity survey, will be collected and managed using Research Electronic Data Capture (REDCap) [32] hosted at The University of Sydney. All other quantitative data, such as recruitment and intervention logs, and physiotherapy assessment forms will be manually recorded in Microsoft Excel spreadsheets by study staff.

Data from the various Web-based platforms used during the ComeBACK trial (such as the Web-based text message service and the ComeBACK websites) will be extracted and/or analysed by the corresponding online tool. For example, Google analytics will be used to analyse the website usage of all groups and text message data will be extracted from the Web-based server into a Microsoft Excel spreadsheet for analysis.

For qualitative data, audio-recordings of interviews and focus groups will be transcribed verbatim and imported into NVivo (version 12, QSR International, Melbourne, Australia) to assist in the process of data analysis. Initially, a subset of transcripts will be independently coded by two researchers using inductive (data driven) and deductive (driven by the PRACTIS framework and the theories underpinning the ComeBACK interventions as outlined in the logic model) approaches to develop initial codes prior to discussion. Codes will then be discussed, and a coding scheme refined and amended prior to the lead author (SW) continuing to code the remaining transcripts. Codes may continue to evolve in response to the data. Thematic analysis will be used to examine the categories of coded data and report on patterns within the data [33]. Divergent views will be recorded in any publications.

In order to more clearly understand the mechanisms relating to the delivery of the ComeBACK interventions, triangulation of the quantitative and qualitative data will occur in order to examine the data from different perspectives. For example, when assessing the fidelity of the *Coaching to ComeBACK* and *Texting to ComeBACK* interventions, data from the logs reporting the number of sessions, duration and content of each session will be analysed in conjunction with interview responses from the health coaches regarding the delivery of the intervention. More aligned responses may help to validate findings about delivery fidelity, whilst variation between the different data sources may prompt further investigation as to the underlying reasons [34].

### Trial status

The ComeBACK trial is currently under way, with recruitment having commenced in February 2019. Recruitment is likely to be completed in 2022.

## Discussion

In this paper, we describe a process evaluation specifically designed to incorporate assessment of implementation and scalability as well as prospective evaluation of two physical activity interventions for adults living in the community with a self-reported walking difficulty. We do this using the structure and framework of the PRACTIS guide, a four-step process which considers the means and suitability of the intervention to real-world implementation at scale. The information gathered from this evaluation will contribute to step 4 of the PRACTIS guide, addressing the barriers to implementation and scale-up.

This process evaluation has a number of strengths. Framed by the PRACTIS guide, it has a structured, comprehensive approach to data collection (qualitative and quantitative) and engagement with key stakeholders across all levels of the system. We believe this provides the first worked example of how this framework may be useful to structure future process evaluations within physical activity trials, in addition to the more formative development of intervention components. The use of mixed methods and multiple data sources will add depth and richness to the findings, allowing for an in-depth qualitative understanding of intervention delivery and implementation processes, backed with quantitative data. The process evaluation will not only report on the implementation of the two ComeBACK interventions, but also assess the various methods used for trial recruitment, providing valuable information on future dissemination specifically targeting this population.

There are also limitations to this process evaluation, one being the risk of reporting bias with a large amount of the data self-reported by health coaches. However, it is envisaged that this will be minimised through triangulation of data from multiple sources. It is unclear at this stage the depth to which we can explore the barriers and facilitators to these types of physical activity interventions with all relevant stakeholders, such as at the level of government policymakers. There is also a broader question not addressed in this evaluation, but which would be relevant to future implementation, which is why people decline to take part in these types of interventions. However, this population can be challenging to engage and (for pragmatic reasons) it is not part of this evaluation.

This process evaluation aims to assist in interpreting the findings of the ComeBACK trial, as well as adding rich information about how the ComeBACK interventions may be successfully implemented. Specifically, it will provide insights into potential barriers and facilitators to intervention delivery, implementation and scale-up of interventions to increase physical activity. It also provides a worked example of how to plan and conduct a process evaluation with a focus on implementation and scale-up.

## Supplementary Information

**Additional file 1.** ComeBACK interview guides.

**Additional file 2.** Impressions of the program questionnaire.

## Data Availability

Not applicable.

## Abbreviations

COM-B: Capability, Opportunity, Motivation–Behaviour model of behaviour change
ComeBACK: Coaching and Exercise for Better Walking (study name)
FRAME: Framework for Modification and Adaptions—Expanded
GP: General practitioner
PACES: Physical Activity Enjoyment Scale
PRACTIS: Practical planning for Implementation and Scale-up
REDCap: Research Electronic Data Capture
SMS: Short Messaging Service
UK MRC: United Kingdom Medical Research Council
