# Supplementary material for: Planning implementation and scale-up of physical activity interventions for people with walking difficulties: study protocol for the process evaluation of the ComeBACK trial
Source: Trials. 2022 Jan 15;23:40. doi: 10.1186/s13063-021-05990-3 (PMC8760869; doi:10.1186/s13063-021-05990-3)
Supplement: Supplementary file 1 — Additional file 1. ComeBACK interview guides. [file 13063_2021_5990_MOESM1_ESM.docx]

**Experiences and attitudes of participants Coaching to ComeBACK: longitudinal interview study**

**Aim:** To describe the expectations, experiences and impact of telephone health coaching for people with walking problems.

**Health Coaching Interview 1 (prior to start of coaching sessions)**

Thank you for agreeing to participate in this part of the study. We are looking forward to hearing about your experience over the next few months as you have your health coaching sessions. This interview today is just to get some information about your thoughts and expectations prior to receiving any coaching sessions. It will help us review whether we are informing people adequately prior to coaching and to assist in the development of health coaching services in the future.

We will be talking about health coaching and physical activity. When we are talking about physical activity, I am referring to things like walking for exercise or to get to and from places, home exercises or exercise groups in the community, sport, and any active tasks you do for work or around the house like washing the floors/mowing the lawns.

Let me know at any stage if you would like me to clarify any of the questions as we go. It's likely to take around will take around 30 - 40mins. I will be recording our conversations and as I mentioned previously your identity will be kept confidential and all of the information gathered from these interviews will be anonymous.

1. Could you tell me a bit about how you came to be involved in this project? /What made you decide to be involved?

**Definition and need for health coaching**

1. When you hear the words ‘health coaching’, what comes to mind for you?
2. What do you believe is the role of a health coach?

**Expectations of heath coaching**

1. What do you think/what would you like the health coaching sessions to include for you?
2. Do you think the health coach will change your level of physical activity – why/why not?

**Potential motivators and barriers**

1. Can you envisage a time in your life when you had a good/challenging experience with coaching/mentoring? Can you describe what sorts of things made this coaching experience a positive/challenging one?
2. If there were one thing you would like to gain from this health coaching experience, what would it be - why?
3. Do you think there will be any challenges – why?

**Wrap up**

1. We are just about finished: is there anything else you would like to add that you think might be relevant?

**Health Coaching Interview 2 (approx. 3-6mths post randomisation)**

Thank you for your involvement in this part of the study. So, this is our first follow up interview since you have started your health coaching sessions. I am interested to hear how you have found the sessions thus far.

So again, like last time, I’m going to go through a number of questions with you today. Let me know at any stage if you would like me to clarify any of the questions as we go. The interview will take around 30 - 40mins. I will be recording our conversations and, as I mentioned previously, your identity will be kept confidential, and all of the information gathered from these interviews will be anonymous.

1. So, to start with, I just wanted to check in and see how the health coaching sessions were going? What are your thoughts about how frequently/how often you have been contacted? Have there been any issues in arranging phone calls?
2. Has the health coaching met your expectations so far - why/why not?

**Appropriate handover**

1. To what degree do you feel that the health coach understood you and your condition when they initially contacted you – why?

**Therapeutic alliance**

1. Do you feel your health coach has been able to listen to you and work with you to achieve your goals - why/why not?

**Intervention**

1. Have you noticed a change in your physical activity since you have had the health coaching sessions – why/why not?
2. Based on the health coaching you have been receiving, what are some of the things you find most valuable/useful for you? And things that you do not find valuable/useful? (refer to booklet, website, coaching, pedometer)
3. Do you feel more confident in being physically active now – why/why not?

**Wrap up**

1. If you had an opportunity to be a health coach for a session, what sort of things would you tell yourself in order to improve your physical activity?
2. We’re just about finished: is there any advice you have for those carrying out the health coaching or those that may be receiving it in the future?

**Health Coaching Interview 3: (approx. 9-12mths post randomisation)**

Thank you again for your involvement in this part of the study. So this is our last follow up interview with regards to this project and at this stage we are really interested in your reflections, thoughts and experiences participating in a health coaching intervention to improve your physical activity. Thank you for all your time and involvement to date and we really value your feedback.

So again, I’m going to go through a number of questions with you today. Let me know at any stage if you would like me to clarify any of the questions as we go. I envisage the interview will take around 30 - 40mins. I will be recording our conversations and, as I mentioned previously, your identity will be kept confidential, and all of the information gathered from these interviews will be anonymous.

1. So firstly, I just wanted to check in and hear how you have been over the past few months since the health coaching has finished?

**Intervention**

1. Have you noticed any changes in your walking function over the past 6 months since the health coaching has stopped? Why do you think these changes have occurred?
2. When reflecting back over the last 12 months since you joined the ComeBACK program, have you noticed any change in your walking function or physical activity? Why do you think these changes have occurred?
3. Do you feel more confident in being physically active now – why/why not?
4. Would you recommend this type of intervention – a 6 month health coaching programme - for other people with walking difficulties who want to increase the amount of physical activity they do – why/why not?

**Potential motivators/barriers**

1. Have there been any challenges in maintaining or improving your physical activity since you have completed the health coaching sessions? Why/Why not?
2. Have you used or can you think of any strategies to overcome these challenges?

**Wrap Up**

1. We’re just about finished: do you have any final comments or thoughts?

**Experiences and attitudes of participants receiving Texting to ComeBACK: longitudinal interview study**

**Aim:** To describe the expectations, experiences and impact of tailored advice and digitally delivered

messaging for adults with walking problems.

**Texting to ComeBACK Interview 1 (prior to start of intervention commencement)**

Thank you for agreeing to participate in this part of the study. We are looking forward to hearing about your experience receiving the text message interventions to support your physical activity over the coming months. This interview today is just to get some information about your thoughts and expectations prior to receiving any tailored advice or text messaging. It will help us review whether we are giving people enough information about the service prior to receiving it. It will also help to assist in the development of these services in the future.

We will be talking about the telephone call of advice from a health coach you will receive and the subsequent text messages encouraging you to be physically active. When we are talking about physical activity, I am referring to things like walking for exercise or to get to and from places, home exercises or exercise groups in the community, sport, and any active tasks you do for work or around the house like washing the floors/mowing the lawns.

It's likely to take around will take around 30 - 40mins. Let me know at any stage if you would like me to clarify any of the questions as we go. I will be recording our conversations and as I mentioned previously your identity will be kept confidential and all of the information gathered from these interviews will be anonymous.

__________________________________________________________________________________

1. Could you tell me a bit about how you came to be involved in this project? / What made you

decide to be involved?

**Need for text message support**

2. What do you believe is the role of text messaging in relation to your physical activity?

**Expectations of single tailored advice and text messages**

3. What do you think or would like the one-off phone call of tailored advice to include? Is there anything you don’t want it to include?

4. What do you think or would like the text messages to include for you? Is there anything you feel you do not want to receive?

5. Do you think this approach, of a one-off phone call with a health coach and motivational text messages will change your level of physical activity – why/why not?

**Potential motivators and barriers**

6. Have you ever had any experience previously of a motivational text message or remote support (e.g. Emails) service that you have been engaged with? What made this experience positive?

Were there any challenging aspects to it? How did you overcome these?

7. If there were one thing you would like to gain from your involvement in this trial, what would it

be? Why?

8. Do you think there will be any challenges along the way? Why?

**Wrap up**

9. We are just about finished: is there anything else you would like to add that you think might be

relevant?

**Texting to ComeBACK Interview 2 (approx. 3-6mths post randomisation)**

Thank you for your involvement in this part of the study. So this is our first follow up interview since you have started your Texting to ComeBACK program. I am interested to hear how you have found the program thus far.

So again, like last time, I’m going to go through a number of questions with you today. Let me know at any stage if you would like me to clarify any of the questions as we go. The interview will take around 30 - 40mins. I will be recording our conversations and, as I mentioned previously, your identity will be kept confidential, and all of the information gathered from these interviews will be anonymous.

__________________________________________________________________________________

1. So to start with, I just wanted to check in and see how the messages were going?

2. Have the text messages met your expectations so far - why/why not?

**Appropriate handover**

3. To what degree do you feel that the health coach understood you and your condition when they initially contacted you? How did this make you feel?

**Therapeutic alliance**

4. Do you feel your health coach, the person who contacted you initially and is responsible for the text messages, has been able to work with you to achieve your goals so far? Why/why not?

**Intervention**

5. Have you noticed a change in your physical activity since you have had the one-off phone call with the health coach and the text messaging – why/why not?

6. Based on the initial session of advice and the text messaging you have been receiving, what are some of the things you find most valuable/useful for you? And what things have you found not find valuable/useful?

7. Have you found the booklet useful? Why/why not? And the website?

8. Do you feel more confident in being physically active now – why/why not?

**Messaging frequency and content**

9. What are your thoughts about how frequently/how often you have been contacted?

10. What are the key messages that have resonated with you? why?

**Wrap up**

11. What sort of content would you put in text message in order to motivate yourself to improve your physical activity?

12. We are just about finished: is there anything else you would like to add that you think might be relevant?

**Texting to ComeBACK Interview 3 (approx. 9-12mths post randomisation)**

Thank you again for your involvement in this part of the study. So, this is our last follow up interview with regards to this project and at this stage we are really interested in your reflections, thoughts and experiences participating in a text messaging intervention to improve your physical activity. Thank you for all your time and involvement to date and we really value your feedback.

So again, I’m going to go through a number of questions with you today. Let me know at any stage if you would like me to clarify any of the questions as we go. I envisage the interview will take around 30 - 40mins. I will be recording our conversations and, as I mentioned previously, your identity will be kept confidential, and all of the information gathered from these interviews will be anonymous.

__________________________________________________________________________________

1. So firstly, I just wanted to check in and hear how you have been over the past few months since the text messages have finished?

**Intervention**

2. Have you noticed any changes in your walking function over the past 6 months since the text messages have stopped? Why do you think these changes have occurred?

3. When reflecting back over the last 12 months, have you noticed any change in your walking function or physical activity? Why do you think these changes have occurred?

4. Do you feel more confident in being physically active now – why/why not?

5. Would you recommend this type of intervention – a phone call of tailored advice and text messaging- for other people with walking difficulties who want to increase the amount of physical activity they do – why/why not?

**Potential motivators/barriers**

6. Have there been any challenges in maintaining or improving your physical activity since you no longer receive any of the messages? Why/Why not?

7. Have you used, or can you think of any strategies to overcome these challenges?

**Wrap Up**

8. We’re just about finished: do you have any final comments or thoughts?

**Experiences and attitudes of stakeholders in the ComeBACK trial – Intervention providers: Health Coaches**

**Aim:** To describe the attitudes and experiences of delivering the ComeBACK interventions for people with walking problems.

Thank you for agreeing to participate in this part of the study. We are looking forward to hearing about your experiences in delivering the ComeBACK interventions over the last 2 years. This interview today is just to get some information about your thoughts and experiences in the delivery of both the Coaching to ComeBACK and Texting to ComeBACK interventions to help inform any future implementation of the interventions.

Let me know at any stage if you would like me to clarify any of the questions as we go. It's likely to take around 45-60 mins. I will be recording our conversations and as I mentioned previously your identity will be kept confidential and all of the information gathered from these interviews will be anonymous.

1. Can you tell me about the training you have undertaken to gain skills in health coaching? What sort of skills has this training given you that are important for a health coach to have?
2. What type of participants responded to the Coaching intervention? Why/why not?
3. What elements of health coaching did you feel resonated most with participants? Why do you think this was? What did not resonate with participants?

**Expectations of the intervention**

1. Prior to commencement of the trial, did you feel that the interventions (*Coaching to ComeBACK* and *Texting to ComeBACK*) would meet the needs of this population? Why/Why not?
2. Did that view change over the course of the trial? How?

**Mechanisms of the intervention**

1. What role does therapeutic alliance play in delivering these interventions?
2. How do you feel participants responded to the ComeBACK booklet? Why do you think this might be?
3. How useful / beneficial was the physiotherapy assessment (paper-based information) for the Coaching to ComeBACK group? Why?
4. How useful / beneficial was the physiotherapy handover/three-way communication with PT/participant and you for your understanding of the participant? In what ways?
5. How adequately did you understand the physical capacity of those in the Texting to ComeBACK intervention (without the handover)? How was this different to the Coaching group?
6. Was the frequency and duration of sessions with the *Coaching to ComeBACK* participants sufficient? Why/Why not?
7. Was the frequency and duration of intervention (considering both the initial tailored phone call and the ongoing messages) with the *Texting to ComeBACK* group sufficient? Was this adequate? Why/Why not
8. How useful was the physical activity plan? Did participants engage with it? Did participants report any dialogue with their GP about the PA plan?
9. Did the use of apps, Fitbit or simple pedometers feature prominently in the interventions? Did they add to the participants experience? How?
10. From your discussions with participants, how did they engage with the ComeBACK website? Why do you think they engaged in this way?
11. From your experience delivering the interventions, are there any components of the two interventions that you would remove/change if you had the choice?

**Barrier and facilitators in delivering the interventions**

1. What were the things that assisted with / facilitated the delivery of the Coaching to ComeBACK intervention? What things made it difficult to deliver? (Prompts: complexity of participants, access to appropriate and affordable community physical activity options)

**Implementation and scale up of the intervention**

1. As part of the trial you needed to screen people into the trial. Would there need to be a screening process into a service delivering this intervention and how could this work?
2. As part of the trial we recruited people directly from health services and people living in the community. Did you see differences in these people and do you think a service delivering these interventions should target both groups of people?
3. What can you envisage might be challenging if delivering this type of intervention at a state or national level? Why?
4. What can you see would facilitate delivery of this type of intervention at a state or national level? Why?

**Wrap up**

1. We are just about finished: is there anything else you would like to add that might help us improve the design or delivery of interventions like ComeBACK?

**Experiences and attitudes of stakeholders in the ComeBACK trial – Intervention providers: Physiotherapists providing assessments for the Coaching to ComeBACK group**

Thank you for agreeing to participate in this part of the study. We are looking forward to hearing about your experiences in delivering the ComeBACK interventions over the last 2 years.

This interview today is just to get some information about your thoughts and experiences in the delivery of assessment in the Coaching to ComeBACK group to help inform any future implementation of the interventions.

Let me know at any stage if you would like me to clarify any of the questions as we go. It's likely to take around 20-30 mins. I will be recording our conversations and as I mentioned previously your identity will be kept confidential and all of the information gathered from these interviews will be anonymous.

1. Could you tell me what was your role involved with the ComeBACK trial?
2. How were you recruited to carry out the assessments?

**Expectations of the intervention**

1. What is your understanding of how your assessment fit into the larger ComeBACK RCT?

**Intervention**

1. Was the physiotherapy assessment that you conducted straightforward and easy to follow?
2. What was the most useful part of the assessment for passing on information about the participants capacity to engage in physical activity?
3. Was there any part of the assessment that you felt was unnecessary? Why/Why not?
4. Did you experience any challenges in delivering this type of assessment to this population? Were you able to overcome them?

**Barrier and facilitators in delivering the interventions**

1. What can you envisage might be challenging if delivering this type of intervention (and 1:1 physiotherapy assessment) at a state or national level? Why?
2. What can you see would facilitate delivery of this type of intervention at a state or national level? Why?

**Wrap up**

1. We are just about finished: is there anything else you would like to add that you think might be relevant?

**Experiences and attitudes of stakeholders in the ComeBACK trial – Implementation and Scale up**

**Aim:** To describe the attitudes of stakeholders who would be potentially responsible for the future implementation and scale up of the ComeBACK interventions for people with walking problems. This will include health service physiotherapists from referral sites, health service managers and other decision makers within the NSW healthcare system.

**Implementation and Scale Up Interviews / Focus groups**

Thank you for agreeing to participate in this part of the study. We are looking forward to hearing about your attitudes about the implementation and scale up of an intervention such as the ComeBACK interventions in the Australian healthcare context.

We are interested in hearing your experiences, thoughts and attitudes towards the barriers and facilitators to implementing interventions like ComeBACK at scale, and how we might mitigate some of those barriers to increase the likelihood of more successful implementation. The ComeBACK interventions consist of the following:

*Coaching to ComeBACK group*: one face-to-face assessment from a physiotherapist, tailored physical activity plan sent to participant and GP, physical activity phone coaching from a physiotherapist, activity monitors and/or apps, booklet and access to on-line resources. *Texting to ComeBACK group*: single session of tailored advice by phone from a physiotherapist with health coaching training, tailored physical activity plan sent to participant and GP, unidirectional text messages, booklet and access to on-line resources.

It's likely that the interviews / focus groups will take around will take around 30-40 mins. Let me know at any stage if you would like me to clarify any of the questions as we go. I will be recording our conversations and as I mentioned previously your identity will be kept confidential and all of the information gathered from these interviews will be anonymous.

1. Could you tell me about the healthcare context / environment in which you are employed and your role within that organisation?
2. The ComeBACK trial is evaluating the effectiveness of two types of interventions to increase physical activity. Do you feel these types of interventions (health coaching and texting) meet the needs of adults living in the community with a self-reported walking difficulty? Why/Why not?

**Expectations of recruitment and the interventions**

1. Recruitment from the end of a health service admission has been challenging during the ComeBACK trial. How do you think we can better engage this group of people at this point in interventions to increase their physical activity?
2. What are some of the factors which influence your organisation in supporting these types of interventions?

**Future implementation**

1. If these types of interventions were shown to be effective, what are the strengths of the interventions that make them more likely to be implemented successfully at scale for?
2. What would be some of the challenges in implementing these interventions at scale? How can these challenges be minimised or addressed?

**Wrap up**

1. We are just about finished: is there anything else you would like to add that you think might be relevant?
