# Supplementary material for: Planning implementation and scale-up of physical activity interventions for people with walking difficulties: study protocol for the process evaluation of the ComeBACK trial
Source: Trials. 2022 Jan 15;23:40. doi: 10.1186/s13063-021-05990-3 (PMC8760869; doi:10.1186/s13063-021-05990-3)
Supplement: Supplementary file 2 — Additional file 2. Impressions of the program questionnaire. [file 13063_2021_5990_MOESM2_ESM.doc]

**Impressions of the Coaching to ComeBACK program at 6 months (Group 1)**

We are interested in your impressions of the physical activity program you have been encouraged to take part in for the last 6 months.

1. Overall, how beneficial do you think the program has been to you so far?

(0 = no benefit & 10 = extremely beneficial)

0 1 2 3 4 5 6 7 8 9 10

No benefit Neutral Extremely beneficial

Why?______________________________________________________________________________________________

_____________________________________________________________________________________________________

1. Would you recommend this type of program to other people with walking difficulties?

Yes No Unsure

If you answered no, why not?_____________________________________________________

_____________________________________________________________________________

1. How would you rate the features of your program?
2. Information package

0 1 2 3 4 5 6 7 8 9 10

No benefit Neutral Extremely beneficial

Why?__________________________________________________________________________

1. Initial physiotherapy assessment

0 1 2 3 4 5 6 7 8 9 10

No benefit Neutral Extremely beneficial

Why?_________________________________________________________________________________

1. Telephone health coaching sessions

0 1 2 3 4 5 6 7 8 9 10

No benefit Neutral Extremely beneficial

Why?__________________________________________________________________________________

1. Website resources

0 1 2 3 4 5 6 7 8 9 10

No benefit Neutral Extremely beneficial

Why?___________________________________________________________________________________

1. Did you use any other technology during your program (eg. Fit bit or exercise apps)?

Yes No Unsure

If you did use a fit bit/activity monitor, how would you rate its benefit?

0 1 2 3 4 5 6 7 8 9 10

No benefit Neutral Extremely beneficial

Why?_______________________________________________________________________________

If you did use exercise apps, how would you rate their benefit?

0 1 2 3 4 5 6 7 8 9 10

No benefit Neutral Extremely beneficial

Why?________________________________________________________________________________

1. Overall, how would you rate your ability to take part in the physical activity program?

(0 = very poor & 10 = excellent)

0 1 2 3 4 5 6 7 8 9 10

Very poor Neutral Excellent

1. Did the program meet your expectations?

Yes No Unsure

Why/Why not?__________________________________________________________________

1. Did you experience any barriers to participation in the program?

Yes No Unsure

If yes please give details:___________________________________________________________

_______________________________________________________________________________

1. Did your General Practitioner (GP) discuss physical activity with you during any of your appointments?

Yes No Unsure

1. Do you have any other comments about the physical activity program?

_______________________________________________________________________________

_______________________________________________________________________________

_______________________________________________________________________________

_______________________________________________________________________________

Thank you for completing this questionnaire. Please return it in the envelope provided

.

**Impressions of the Texting to ComeBACK program at 6 months (Group 2)**

We are interested in your impressions of the physical activity program you have been encouraged to take part in for the last 6 months.

1. Overall, how beneficial do you think the program has been to you so far?

(0 = no benefit & 10 = extremely beneficial)

0 1 2 3 4 5 6 7 8 9 10

No benefit Neutral Extremely beneficial

Why?______________________________________________________________________________________________

_____________________________________________________________________________________________________

1. Would you recommend this type of program to other people with walking difficulties?

Yes No Unsure

If you answered no, why not?_____________________________________________________

_____________________________________________________________________________

1. How would you rate the features of your program?
2. Information package

0 1 2 3 4 5 6 7 8 9 10

No benefit Neutral Extremely beneficial

Why?__________________________________________________________________________________

1. Telephone physiotherapy advice

0 1 2 3 4 5 6 7 8 9 10

No benefit Neutral Extremely beneficial

Why?__________________________________________________________________________________

1. Website resources

0 1 2 3 4 5 6 7 8 9 10

No benefit Neutral Extremely beneficial

Why?___________________________________________________________________________________

1. Text messaging service

0 1 2 3 4 5 6 7 8 9 10

No benefit Neutral Extremely beneficial

Why?_______________________________________________________________________________

1. Overall, how would you rate your ability to take part in the physical activity program?

(0 = very poor & 10 = excellent)

0 1 2 3 4 5 6 7 8 9 10

Very poor Neutral Excellent

1. Did the program meet your expectations?

Yes No Unsure

Why/Why not?__________________________________________________________________

1. Did you experience any barriers to participation in the program?

Yes No Unsure

If yes please give details:___________________________________________________________

_______________________________________________________________________________

1. Did your General Practitioner (GP) discuss physical activity with you during any of your appointments?

Yes No Unsure

1. Do you have any other comments about the physical activity program?

_______________________________________________________________________________

_______________________________________________________________________________

_______________________________________________________________________________

_______________________________________________________________________________

Thank you for completing this questionnaire. Please return it in the envelope provided.

**Impressions of the Texting to ComeBACK Later program (Group 3)**

We are interested in your impressions of the physical activity program you have been encouraged to take part in for the last 6 months.

1. Overall, how beneficial do you think the program has been to you so far?

(0 = no benefit & 10 = extremely beneficial)

0 1 2 3 4 5 6 7 8 9 10

No benefit Neutral Extremely beneficial

Why?______________________________________________________________________________________________

_____________________________________________________________________________________________________

1. Would you recommend this type of program to other people with walking difficulties?

Yes No Unsure

If you answered no, why not?_____________________________________________________

_____________________________________________________________________________

1. How would you rate the features of your program?
2. Information package

0 1 2 3 4 5 6 7 8 9 10

No benefit Neutral Extremely beneficial

Why?__________________________________________________________________________________

1. Telephone physiotherapy advice

0 1 2 3 4 5 6 7 8 9 10

No benefit Neutral Extremely beneficial

Why?__________________________________________________________________________________

1. Website resources

0 1 2 3 4 5 6 7 8 9 10

No benefit Neutral Extremely beneficial

Why?___________________________________________________________________________________

1. Text messaging service

0 1 2 3 4 5 6 7 8 9 10

No benefit Neutral Extremely beneficial

Why?_______________________________________________________________________________

1. Overall, how would you rate your ability to take part in the physical activity program?

(0 = very poor & 10 = excellent)

0 1 2 3 4 5 6 7 8 9 10

Very poor Neutral Excellent

1. Did the program meet your expectations?

Yes No Unsure

Why/Why not?__________________________________________________________________

1. Did you experience any barriers to participation in the program?

Yes No Unsure

If yes please give details:___________________________________________________________

_______________________________________________________________________________

1. Did your General Practitioner (GP) discuss physical activity with you during any of your appointments?

Yes No Unsure

1. Do you have any other comments about the physical activity program?

_______________________________________________________________________________

_______________________________________________________________________________

_______________________________________________________________________________

_______________________________________________________________________________

Thank you for completing this questionnaire. Please return it in the envelope provided.
